# Community engagement in research in sub-Saharan Africa: current practices, barriers, facilitators, ethical considerations and the role of gender - a systematic review

**DOI:** 10.11604/pamj.2022.43.152.36861

**Published:** 2022-11-23

**Authors:** Luchuo Engelbert Bain, Claudine Akondeng, Wepnyu Yembe Njamnshi, Henshaw Eyambe Mandi, Hubert Amu, Alfred Kongnyu Njamnshi

**Affiliations:** 1 Triangle Research Foundation (TRIFT), Limbe, Cameroon,; 2 Department of Psychology, Faculty of Humanities, University of Johannesburg, Johannesburg, South Africa,; 3 Global South Health Services and Research (GSHS), Amsterdam, The Netherlands,; 4 Brain Research Africa Initiative (BRAIN), Yaoundé, Cameroon,; 5 Cameroon National Association of Family Welfare (CAMNAFAW), Yaoundé, Cameroun,; 6 Education and Learning for All (ELFA) Cameroon, Yaoundé, Cameroon,; 7 Division of Operational Research in Health, DROS, Ministry of Public Health, Yaoundé, Cameroon,; 8 Coalition for Epidemic Preparedness Innovations, Oslo, Norway,; 9 Department of Population and Behavioral Sciences, Fred N. Binka School of Public Health, University of Health and Allied Sciences, Hohoe, Ghana,; 10 Faculty of Medicine and Biomedical Sciences (FMBS), The University of Yaoundé I, Yaoundé, Cameroon

**Keywords:** Research, ethics, gender, sub-Saharan Africa, barriers and facilitators, community engagement

## Abstract

**Introduction:**

meaningful community engagement is increasingly being considered the major determinant of successful research, innovation and intervention uptake. Even though there is available literature recommending community engagement in health research, there are still knowledge gaps in how communities might be best engaged in Sub-Saharan Africa. We, therefore, synthesized the existing literature on the current practices, barriers and facilitators, ethical considerations, and gender mainstreaming in the engagement of communities in research in sub-Saharan Africa.

**Methods:**

this synthesis was developed following the Preferred Reporting Items for Systematic review and Meta-Analysis (PRISMA). A combination of keywords and medical subject headings was used to search MEDLINE, EMBASE, Global Health Library through OVID SP, the Cochrane Library, PsychINFO, CINAHL, WHO Afro Library, WHO Global Index Medicus and the National Institute for Health Research, for all literature published between 1 January 2000 to 31 July 2021.

**Results:**

thirty articles met our inclusion criteria. The key reported facilitators of effective community engagement in research included appropriate community entry and engagement of stakeholders. Barriers to effective community engagement in research included the availability of prohibitive cultural, historical and religious practices; geographical/spatial limitations, difficulties in planning and executing community engagement activities and communication barriers. Awareness creation and sensitization on the research through drama, social media, documentaries, and community durbars are some of the existing practices adopted in engaging communities in research. Gender mainstreaming was not considered appropriately in the engagement of communities in research, as only a few studies made provisions for gender considerations, and most of the time, interchanging gender for sex. Respect for autonomy, privacy and informed consent were the main ethical issues reported.

**Conclusion:**

gender mainstreaming and ethical standards were reported as important, but not explored in depth. Gender as a social construct needs to be carefully integrated in the entire research cycle. Clear ethical concerns within a research project have to be co-discussed by the research team, community members and potential research participants.

## Introduction

Community engagement (CE) in research can be defined as a process of inclusive participation that supports mutual respect of values, strategies and actions for the authentic partnership of people affiliated with or self-identified by geographic proximity, special interest, or similar situations to address issues affecting the well-being of the community of focus [1]. Meaningful CE is increasingly considered the major determinant of the successful uptake of research, innovation and intervention. Community leaders, policymakers and funders have expressed the need to engage communities in research [1]. National and international bodies now recognize the importance of CE in research. However, CE guidelines are arguably unclear, making their implementation and evaluation difficult, potentially leading to missed opportunities, squandered resources, and poor decisions [2]. Community engagement in research encourages the host community to participate in addressing its own health needs and ensures that researchers understand community priorities [1]. Indeed, existing evidence suggests that building trust and ownership of the research endeavour among stakeholders, especially the host community for the research project, is ideal to guarantee success and sustainability [3,4]. In 2009, the Human Immunodeficiency Virus Prevention Trials Network´s (HPTN) ethics guidance for research, directly addressed CE as an ethical obligation in guidance point 3: “in order to ensure that HPTN research is appropriate as well as scientifically and ethically sound, relevant communities will be engaged in a meaningful process that will help guide the research from protocol development to dissemination of results” [2]. Thus, effective CE strategies have to be carefully integrated into the design, implementation, monitoring and sustainability phases of the research process [5].

Ethical CE entails upholding high ethical standards. When we engage the community, we make certain that marginalized or disempowered subgroups in the community are included in the research project [6]. This has the potential to reduce trial attrition rates and improve uptake of trial outcomes. In addition, CE facilitates the community´s willingness to participate in future trials and other healthcare interventions [7]. Appropriate CE promotes co-production, co-implementation and co-evaluation, which may strengthen both the sense of inclusion, ownership, and the effectiveness of the research life-cycle [4,8]. In contrast, an inappropriate CE strategy affects the project under investigation and jeopardizes the trust between the research team and the community in question.

Different organizations and research bodies use diverse strategies and protocols to engage communities in research activities. However, there is little information on the role gender plays in effective CE, thus justifying the need to assess gender dynamics across the entire spectrum of the research process in African communities [9]. Gender-sensitive community engagement can be defined as approaches to engaging communities that promote gender equality and empowerment and respect existing context-specific gender norms [9]. Developing guidelines of best practices to guide researchers in engaging communities in research activities in a gender-sensitive way in Sub-Saharan African countries is imperative, as it stands out to be a key contribution to attaining the sustainable development goal 5 on gender equality [10].

The rise of community-based participatory research (CBPR) suggests that researchers are becoming more embedded in the communities they are studying and are committed to preventing harm and promoting social justice while conducting research, as well as developing caring relationships. Thus, an awareness of the potential complexities and conflicts and a willingness and ability amongst research collaborators to reflect on such ethical issues throughout the planning and conduct of CBPR are fundamental [7,11]. Culturally-specific community engagement in research necessitates understanding the perspectives of diverse populations and disciplines and applying that understanding to the process of involving communities in a research project (for instance, culturally-appropriate language) [12]. Sustainable CE in research will imply engagement that does not focus solely on the research goals without developing a plan for capacity building and creating a sustainable system that will outlive the research funding period. Actively planning for and developing community capacity to ensure sustainability is especially important in research involving underserved populations and underrepresented minorities [13].

Despite the available literature recommending CE in health research [4,5], there are still knowledge gaps in how communities might be best engaged in SSA [4,14]. Indeed, CE is mentioned in published research to describe community entry, consent and study participant retention. However, the actual CE activities are generally not well documented [15]. Also, the fact that communities are heterogenous suggests that CE activities will not produce similar results across different contexts [15]. In a sub-region where there is great community heterogeneity in terms of culture, our review sought to synthesize available evidence across different countries in SSA on various practices for engaging communities in research and facilitators and challenges to this process. Specifically, this review sought to describe the current practices in involving communities in research, ascertain the barriers and facilitators in effectively engaging communities in research, explore the ethical considerations required in engaging communities in research, and ascertain current trends on how gender is taken into account when engaging communities in research. We adopted the research concept as a cycle, where the available best practice guidelines shall inform how communities should be involved in the planning, execution, termination, and data dissemination phases (publications and conference presentations) of the research project. Our findings could be important in informing policymakers across the sub-region on appropriate factors to consider when engaging communities in the conduct of research.

## Methods

**Figure 1:**
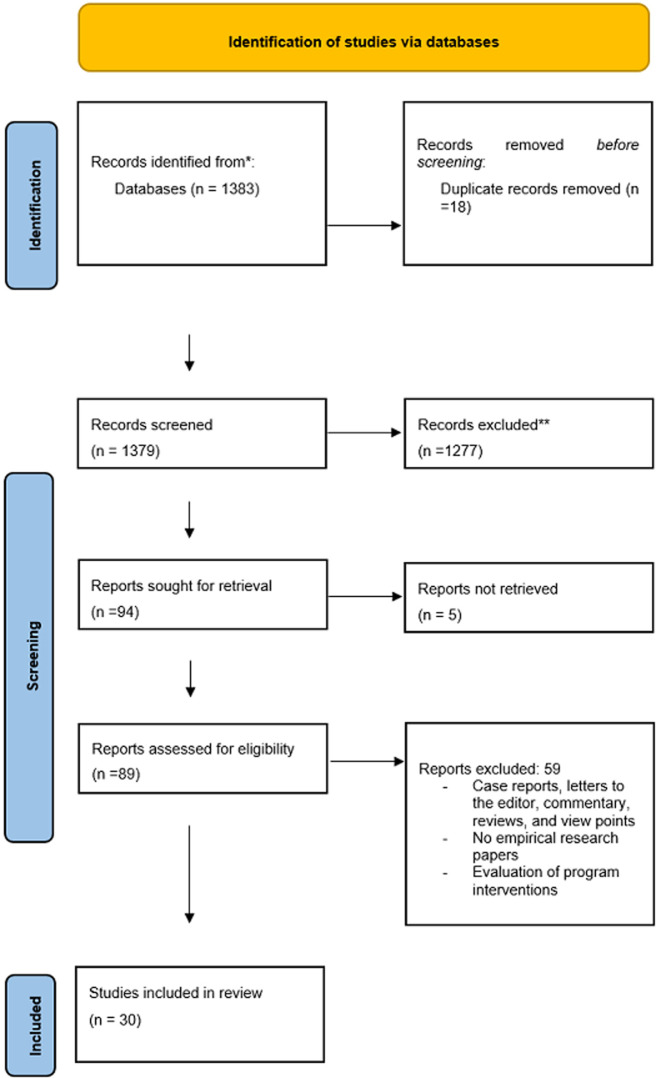
PRISMA flow diagram of included studies

To select the studies, we used a combination of keywords and medical subject headings such as “Community Engagement” or “Community Involvement” were used to search 09 databases for all literature published between 1 January 2000 to 31 July 2021. We searched the following databases: MEDLINE, EMBASE, Global Health Library through OVID SP, the Cochrane Library, PsychINFO, CINAHL, WHO Afro Library, WHO Global Index Medicus and the National Institute for Health Research. Commentaries, personal views, and letters to editors were, however, excluded. Citations retrieved from database searches were exported into EndNote X9 to remove duplicate citations and imported into Rayyan QCRI for screening. The protocol for this systematic review was developed following the Preferred Reporting Items for Systematic Review and Meta-Analysis (PRISMA) [16]. A PRISMA flow diagram for the articles selected (Figure 1), as well as the search strategy (Annex 1) are attached as supplementary files. The articles included had to meet the inclusion criteria, and were both from peer-reviewed articles and grey literature. The inclusion criteria were: articles published between 2000 and 2021, contained research on CE or their implementation involving engaging communities. We were specifically interested in issues around gender, ethics, and best practices when engaging communities in research. We included articles published in the English and French languages.

Two authors (LEB and CA) independently screened citations retrieved from database searches based on title and abstract. The titles and the abstracts were screened by applying the previously stated eligibility criteria before assessing the full texts of selected articles for final inclusion in the review. Disagreements between review authors during the screening stage were resolved through discussions and consensus. In the case of non-resolution, a third reviewer was to serve as a tiebreaker. However, this option was not utilized as such disagreement did not occur. The articles were read extensively, and a thematic analysis was carried out. The major codes and themes were summarized in an Excel file. The six-step thematic approach proposed by Braun and Clarke [17] guided the data analysis process.

**Quality of evidence:** Annex 2 presents the methodological quality assessment of the 30 papers [9,15,18-45] included in our synthesis. This was conducted using a range of critical appraisal tools. Critical appraisal checklists for RCTs [46], qualitative [47] and mixed methods [48,49] studies were used. These tools assessed the methodological quality of the studies and ascertained the extent to which the studies have addressed the likelihood of bias in their design, procedures and analyses.

**Disclaimer:** the lead author (guarantor of the manuscript) certifies that the manuscript is an honest, transparent, and accurate account of the research being reported; that no important aspects (objectives) of the study have been omitted; and that any deviations from the study as planned (and, if relevant, registered) have been explained.

## Results

### Search results

A total of 1383 articles were identified from searching the various databases. The titles and abstracts of these 1383 were reviewed and 89 were retained to be within our scope. A full-text review of the 89 articles was conducted and 30 of them met our inclusion criteria. Figure 1 is a PRISMA flowchart which summarizes the study selection processes. The 30 papers had high quality ranging from 65% to 100%.

### Characteristics of included studies

The characteristics of studies included in the final analysis are listed in Annex 3 and Annex 4. Annex 3 presents studies included that were conducted in single site (country) while the Annex 4 presents studies included that were conducted in multiple sites. Regarding the single-country studies, twenty-one studies were identified. Three of the studies were conducted in Zambia, two in Ghana and Nigeria respectively, six studies were conducted in South Africa, four in Malawi and one in Swaziland, Eswatini, Kenyan and Zimbabwe respectively. The studies were conducted between 2003 and 2020. Nineteen of the studies were qualitative designed studies while two were systematic review studies. Also, regarding the multiple country studies, nine studies were identified and these studies were conducted between 2010 and 2018. All of these studies were qualitative approach studies using in-depth interviews or focus groups discussions. The studies were carried out in Botswana, Cameroon, Ethiopia, Ghana, Kenya, Mali, Nigeria, South Africa, Uganda and Zimbabwe; Burkina Faso, Mali and Tanzania; Thailand, India, South Africa and Canada; Zambia and South Africa; South Africa and Zimbabwe; Botswana, South Africa, and Zimbabwe.

### Findings

Annex 5 presents the themes and sub-themes realized from our qualitative synthesis. Ten out of the 30 studies included in our review reported on current practices in involving communities in research. Two main themes came up based on current practices. They were: awareness creation and sensitization on research co-creation. Fifteen studies also reported on barriers to effective community engagement in research. The barriers reported were prohibitive cultural, historical and religious context, geographical/spatial limitation, ineffective stakeholder engagement, difficulty in planning and executing community engagement activities, community expectations, communication barriers, inadequate representativeness of community stakeholders, logistical challenges, inadequate priorities and negative experience from previous studies. Twenty-two studies reviewed reported on facilitators of effective community engagement in research. The themes reported were: effective community entry and engagement of stakeholders at beginning of the research, effective community engagement strategies, community empowerment, and community motivation. Twelve studies also reported on ethical considerations in engaging communities in research. The themes reported included: cultural considerations, community autonomy, review of research protocols, informed consent, avoidance of community exploitation, ensuring fidelity, risk and benefit, and privacy and confidentiality. Four studies reported on current trends in how gender is taken into account when engaging communities in research. The themes of interest were gender and consent, gender of field workers, and gender equality.

### Current practices in involving communities in research

Regarding awareness creation as a current practice of community engagement in research, seven sub-themes were reported. They entail the use of drama, use of social media, use of documentaries and digital storytelling, use of open community forums, use of community durbars, use of mobile vans with key messages, and use of leaflets. The proliferation of social media platforms has resulted in much easier and faster information dissemination. They are also effective at influencing people's opinions. As a result, researchers use its effectiveness to disseminate research information. Three of the studies [26,30,44] reported on the use of social media. The authors noted that social media serves as a larger medium for reaching a larger population which justifies its adoption. Meiring and colleagues [38] use mobile vans with key messages while Asante and colleagues [34] reported the use of leaflets as means to increase awareness among members of the community during the engagement process.

Community capacity building under the theme of co-creation was noted by 4 of the studies as the current practice of community engagement [20,22,29,31]. The authors noted that training of committee members and community volunteers on the research project, as well as the use of selected community members as co-researchers on the research projects, could play a major role in addressing doubts regarding the research and the institutions involved thereby facilitating community engagement since the people will feel being part of the projects. Involvement of community members in selecting village committees and research volunteers, as well as their contributions to protocol and study instrument development, were reported as important current practices of engaging communities in research projects. Six (06) studies also showed that the use of Community Advisory mechanism (Formation of Community Advisory Boards [CABs]) and Community Liaison Officers (CLO) to facilitate relationships and communication between the research teams and communities are important current practices in engaging communities to participate in research projects [20,24,26,28,30,43].

### Barriers and facilitators of effective community engagement in research

#### Culture and trust

Concerning the prohibitive cultural, historical and religious context, three sub-themes were reported from seven of the publications included in the review [18,33-35,37,43,44]. These comprise restrictions on visiting the homes of newborns, religious ideologies, and mistrust regarding the research. Cultural practices such as restrictions on visiting the homes of newborns by strangers with specific characteristics including women that are menstruating or men who had recently has sexual intercourse could hinder effective community engagement [9,44]. Community mistrust regarding the research projects and suspicions of research by institutions (universities) was reported in five of the studies. Bad past experience of historical colonial exploitation by the white colonial masters and researchers raises scepticism when it comes to trusting the intentions of researchers. Geographical /spatial limitations were noted as barriers in effectively engaging communities in some sites [18,34,37,43].

### Ineffective stakeholder engagement

Two sub-themes emerged in terms of ineffective stakeholder engagement [35,39,40]. These include obtaining consent from study participants without first seeking permission from the community´s leader(s) and failing to conduct proper community entry by researchers. Failing to meet with community leaders and other stakeholders at the start of the research made it difficult to obtain effective community participation in the research. Three sub-themes were also realized under unmet community expectations as a barrier to community engagement. These sub-themes were raising expectations of the community to levels beyond what the proposed research may be able to address, monetary expectations (lack of monetary incentives) and unmet expectations from the previous research experience. Monetary expectations by community members from the research team before participation in the research project were noted by four studies as a barrier to their participation in the project.

### Communication barriers

Concerning communication barriers, seven sub-themes were reported [35,40,43,45]. These were language barriers in communicating important scientific concepts to the community, misinformation, language and literacy barrier in obtaining informed consent, the misconception that inhibits effective communication, limited understanding of health research and lexicon challenges, low literacy levels of community members to fully comprehend the nature or concepts of the research and inadequate feedback to the community by the research team. The language barrier on the part of researchers to effectively communicate important scientific concepts to communities was, for instance, reported by three studies. Also, the low literacy levels of community members to fully comprehend the nature or concepts of the research that is communicated to communities by the research team was a commonly reported theme. A core issue reported was the research teams “unconsciously overpromising” community members of their deliverables.

### Bad memories from previous research

Three sub-themes entailing failure of previous research to achieve their intended objective, personal questions that communities are not comfortable with from previous research experience, feeling disappointed not being included in the research (e.g. trials accompanied by incentives to participate), feeling of researchers not meeting the expectations of the community [33,41].

### Prioritization inadequacies [20,39]

Concerning inadequate priorities, two sub-themes were realized. These comprise competing programs and conflicting agendas. Two studies also argued in their respective studies that when there are multiple research projects running concurrently in communities by different research teams, communities tend to attach different priorities to the various studies which can hinder their willingness and actual involvement in some of the studies.

### Facilitators of effective community engagement in research

Effective community entry and engagement of stakeholders at beginning of the research was reported by nine of the studies included in our review [18,34,35,37,39-41,43,45]. These findings are summarized in Annex 5. The theme had six sub-themes which comprise engaging first, traditional and religious leaders, engagement of community-level political leaders, engagement of communities during protocol formulation stages, engagement of civil society organizations, social mapping, and paying close attention to clear channels of authority in the local communities.

### Role of traditional, political, and religious authorities

Engaging the major traditional and religious authorities were considered key, and should be done from the very start of the research cycle [34,39-41,43]. It was considered important to engage community-level political leaders who are the government representatives in the communities [40,41,43]. The need to engage civil society organizations in the communities as part of the initial stakeholder engagements was also emphasized by one of the studies. Social mapping at the beginning of the research is essential in the identification of pre-existing features of the community including decision-making hierarchies. Social mapping, when done well, even ensures that key stakeholders in the community are effectively identified and engaged [34,39-41]. Concerning effective community engagement strategies, 11 sub-themes were reported [18,34,35,37,39-41,43,45]. These were two-way communication with communities to discuss issues that may be of concern to them, openly acknowledging the role that the entire community plays in the research, utilization of strong communication skills, good cultural knowledge by researchers, effective use of existing social structures and networks in the communities, adding fun to all activities and use of participatory methods, use of new technologies and computers in engagement, holding of regular feedback meetings with stakeholders, carrying out targeted sensitization to ensure that community members understand the research objectives and deliverables, active involvement of the community in decision-making, and creation of community fora such as ‘participative spaces’ and/or ‘participatory workshops’.

Effective community engagement could be facilitated by ensuring community mobilization through targeted sensitization campaigns to ensure that community members understand the research objectives and deliverables. The utilization of strong communication skills through the adoption of social marketing strategies and the use of new technologies including computers also help to facilitate community engagement in research [35,41,43]. With community empowerment [37,39,40], four sub-themes were reported: training and recruitment of local staff as research assistants on research projects in communities was reported one as of the studies. This facilitates the implementation of the entire research project in that as these people are part of the communities and their population could trust them, doubts that communities may have towards the projects may reduce. The formation of community advisory boards (CAB) whose task is to serve as a watchdog over the research and its relationship with the community. The authors also consider the CAB as an important mechanism that constitutes a link between the communities and research institutions and, therefore, serves as the liaison between the community and the research team. The other sub-themes reported under community empowerment were the involvement of marginalized groups and building a strong collaboration with the local information health service department.

### Ethical considerations required in engaging communities in research [18,19,21,24,28,34, 35,37,40,43,45]

Regarding cultural considerations, three sub-themes emerged from our synthesis. These include gaining understanding of community perspectives, beliefs, cultural values, and practices, as well as adopting culturally appropriate ways to communicate research information to communities. The theme also includes the fact that studies involving difficult and complex topics need to be evaluated for their effectiveness in the cultural context within which such studies are being conducted. Regarding the adoption of culturally appropriate ways to communicate research information to communities, for instance, three of the studies argued that there should always be respectful of local community concerns and norms during their engagement for the conduct of research.

Concerning community autonomy as an ethical consideration during community engagement, five sub-themes were realized. These comprise the need for the autonomy of communities in decision making to be enhanced, Community Advisory Boards (CAB) being able to carry out their functions independently of the research team in order to protect the community from any unethical research practices, avoidance of structural coercion, avoidance of the use of coercive methods in referral or recruitment of participants, and a need for research involving young people to be evaluated for their effectiveness. Respect the “autonomy” (identity)of communities during decision-making regarding the engagement of the communities in research. This is because, in communities where the majority of people are poor and access to healthcare is limited, researchers sometimes easily persuade community members to participate in research projects by promising free healthcare which ensures that participants do not question the research processes or objections. Another study indicated that Community Advisory Boards (CAB) which are normally formed to serve as watch dogs over the communities, should be able to carry out their functions as gate keepers of the community in independently protecting the communities from unethical research practices.

Concerning the informed consent, two sub-themes were reported. These entail a need to obtain community-informed consent before engagement and ensuring appropriate consenting processes in populations with low literacy or limited exposure and experience with research. The need for research institutions to obtain informed consent from communities before engagement in research was considered as a core consideration. Exploitation was one of the main themes realized under ethical practices in engaging communities in research. Two sub-themes were realized from this theme. These entail exploitations of locally recruited researchers through unfair employment practices and power imbalance and dynamics between researchers and communities. Power imbalance and dynamics between researchers and poorer communities could lead to undue inducements which influence communities to participate in studies without fully understanding the risks and benefits, as they are attracted to monetary incentives and healthcare that the projects may come with.

Regarding risk and benefit as an ethical practice during community engagement, two sub-themes emerged. These were providing adequate information on research goals, risks, and benefits and avoiding research-related harm to the communities. With regard to providing adequate information on research objectives, risks, and benefits to the communities before engagement, benefits and compensations that come with the research project participation need to be made clear to communities before their engagement. Research-related harm needs to be avoided by researchers when engaging communities in research projects as this is usually the situation. The authors stressed that researchers and research institutions should ensure safety of communities when conducting study activities in communities.

### Gender considerations in engaging communities in research [21,34,40,44]

According to two of the studies [40,44] which reported on the impact of gender on consent decision-making, husbands predominately influenced consent decisions in situations where their wives were participants because of the structure of traditional families, where men are the heads. In light of this, the authors admonished that research involving women should involve their husbands in consent decision-making. Thus, the husbands should be informed of the study aims and possible outcomes as well as any risks and benefits to the study participant. Once this is done, they would not prevent their wives from participating in such research. The gender of community research field workers was reported by one of the studies [21]. The gender of field workers has influences on the engagement process as it determines the extent to which participants feel free to express themselves. The involvement of a particular field worker (male) in a research project involving the opposite gender could expose them to sexual vulnerabilities. As such, the gender of interviewers vis-à-vis the study participants should be considered when assigning data collection during research. Concerning gender equality, three sub-themes came up. These were: the need for both genders to be present at stakeholder meetings, avoidance of c ultural tensions regarding researchers working closely with women, and avoidance of gender-biased research agenda (over concentration of research on women [too much concentration on women at the expense of the health of the men]). The presence of both males and females at community engagement meetings is very crucial in ensuring the success of the research, especially when the research involves women [21,34,44]. However, they point out that cultural tensions could erupt when researchers are perceived to be too close to the women and ask some sensitive questions without the presence of their husbands.

## Discussion

This systematic review explored current practices, the barriers and facilitators, ethical considerations, and gender mainstreaming in engaging communities in research. A total of 30 publications were reviewed to generate qualitative themes and sub-themes. Two arose based on current practices in involving communities in research. They are awareness creation on the research and co-creation. Prohibitive cultural, historical and religious context, geographical/spatial limitation, ineffective stakeholder engagement, difficulty in planning and executing community engagement activities, community expectations, communication barriers, inadequate representativeness of community stakeholders, logistical challenges, inadequate priorities, and negative experience from previous studies were main barriers to effective engagement of communities in research. The facilitators entail effective community entry and engagement of stakeholders at beginning of the research, effective community engagement strategies, community empowerment, and community motivation. Ethical considerations in community engagement had to take into consideration the cultural specificities of respective communities, community autonomy, review of research protocols, informed consent, avoidance of community exploitation, ensuring fidelity and trust, proper communication regarding risks and benefits, and privacy and confidentiality.

Co-creation of project through recruitment and training of community volunteers using mostly youths as co-researchers on the research projects could play a major role in building a community feeling of ownership of the projects, therefore addressing doubts that could have existed regarding the research and the institutions involved [50]. The role of co-creation in community engagement as observed in this review is consistent with the realizations made in other previous studies which indicated that community capacity building and empowerment leads to successful research project outcomes as it boosts community ownership of the project goals and empowerment in project implementation [51-54]. Community awareness creation through using social media, public forums, use of communication vans and leaflets were realized as current practice utilized by research institutions/teams in effectively and efficiently engaging communities in research projects. This current practice of awareness creation could contribute to increasing knowledge and understanding of indigenous communities which might have inadequate research literacy or experiences. Therefore, this enlightenment could serve as a mechanism for strengthening community engagement and uptake of the research project [55]. The findings in this study are congruent with previous studies that also noted the importance of community awareness creation in increasing community participation in a research project and implementation [56-58]. We found in the review that many factors could serve as barriers to effective engagement of communities in research. We report that some prohibitive cultural, historical and religious contexts including restriction on visiting homes of newborns, mistrust regarding the research and suspicious of research institution and religious ideologies of communities affect effective community engagement. These findings are consistent with findings from previous studies which also noted that historical colonial exploitation or past exploitation by researchers constitute a barrier to effective community engagement [59-62]. Community mistrust of researchers for instance could be attributed to past negative experiences of communities of historical colonial exploitation by colonial masters or historical exploitation by academic researchers in the past, therefore, making them skeptical in accepting or participating in research studies for which researchers are recognized to have come from western countries.

Geographical/spatial limitation was identified in our review as a barrier that affects effective community engagement. This observation is congruent with previous studies that noted similar observations [63,64]. We noted that dispersed nature of communities over a large geographic area mostly hinders the effectiveness of community engagement. Also, the poor road networks between communities constitute another barrier that impacts community engagement. For example, the road network in the majority of communities in low- and middle-income countries are poor, therefore engaging communities that live beyond those deplorable and unmotorable roads could become very difficult for the researchers [65-67]. We found in this study that the failure of researchers to meet community leaders at the beginning of the research or failure to do proper community entry impacts effective community engagement. This observation made in this review is consistent with what was realized in existing literature [68,69]. These findings in our review stress the importance of effective community entry at the beginning of research as a key step to effective community engagement [70]. Two-way consultation (community leaders and the population) at all stages of the community engagement process by the researchers is critical and vital to improve the likelihood of community members to participate in the project [71-73]. Lack of monetary incentives and unmet expectations from previous research in communities constitute barriers that affect the commitment of the communities to participate in subsequent research. This observation is congruent with similar findings made in previous studies [74-79]. Our review found that the failure of previous research to achieve intended objectives constitutes a barrier to community engagement in subsequent research projects. The current observation is consistent with observations made in previous studies [80,81]. This could be attributed to the fact that the communities which previously participated in a research project that failed to either sustain or achieve their aims will be discouraged from engaging in new projects since the past ‘deceptive’ experience creates in the mind of the people the sense that the story will be the same, therefore, there is no need to participate.

Despite the barriers highlighted in our review, we observed that there were several facilitators that promote community engagement in research. These were noteworthy: effective engagement of stakeholders at beginning of the research, culture sensitive community engagement strategies, and community empowerment and community motivation. Engaging key stakeholders especially the traditional and religious leaders and community-level government representatives at the beginning of the research projects by research institutions or individuals is pivotal for the success of any research enterprise. In settings in SSA where local leaders (traditional and religious) are much revered by the general populace or their followers, meeting with community and political leaders who are in charge of the governing of communities is very crucial for the success of the engagement. This could be ascribed to the influence the leaders have in facilitating relationship and trust building between the research institution/team and the population therefore providing fertile ground for effective engagement [18,82-88]. Community leaders were known to be most revered and feared in the communities and therefore their engagement during the preparation of the project could facilitate the easy mobilization of the general populace of the communities that are under the control of these leaders. Therefore, their early engagement could lead to the acceptability and feasibility of the research and consequently improving the overall research quality and chance of its success [89-91].

The employment of effective community engagement strategies including two-way communication with communities (leaders and community members), openly acknowledging the role the entire community plays in the research, good cultural knowledge by researchers, effective use of existing social structures and networks in the communities facilitate community mobilization and engagement in the research. This finding of our review is compatible with findings from previous studies that urge researchers to employ effective engagement strategies to conquer the trust of the communities and facilitate community engagement [92-94]. Dyer *et al*. [52] reported that two-way communication at all stages of the community engagement process by researchers is essential in building on mutual respect and clarity of roles and responsibilities of both community leaders and the community members. Another important facilitator realized in our review is community empowerment. The capacity building of local community residents especially the youth through their recruitment and training to serve as research assistants on the projects facilitates the community engagement process. Since these individuals are the integral part of the communities, their direct involvement in the research project could improve the trust level of the community members (strong influence capability) thus reducing all skepticism surrounding the projects or institutions involved. This finding is consistent with previous studies which also indicated the vital role community empowerment can play in community engagement [95-98].

Formation of community advisory boards (CAB) was also realized in our review as an important mechanism that facilitates effective community engagement. The CAB serves as a crucial liaison that facilitates communication and relations between the communities and the research institutions, therefore facilitating community engagement. CAB serves as community watchdog that help to identify community priorities and interests; identify community members to serve on project steering committees and promote community support for and involvement with research [99,100]. We observed in our review that there were some essential ethical considerations that are pre-requisite for community engagement. Paramount among these ethical issues are: ensuring the autonomy of the communities, effective review of protocol, informed consent, fidelity, risk and benefit, and privacy and confidentiality, and avoidance of exploitation. Autonomy of community members to freely choose either to participate or not in research for instance, is one very delicate ethical consideration that researchers or institutions need to respect when trying to engage the communities in research projects. The respect of the autonomy of the community members in the decision-making for participation in research is especially imperative in communities where majority of community members are poor or illiterate, or in settings where healthcare is often limited [101-103]. This is imperative because in communities where resources are very limited or access to healthcare is limited, researchers or institutions could easily coerce vulnerable populace to participate in the research projects with the promise of free healthcare or monetary remuneration, leaving the people with no option to evaluate the risk of partaking in the research. With regards to ensuring that communities are protected from exploitation by the powerful researchers or institutions, the Community Advisory Board (CAB) that serves as a watch dog over the communities´ rights and pursues the welfare and fairness for the community members, should be able to carry out its functions of gate-keeping independently of the research team in order to protect the community from any unethical research practices [104-106]. We realized that structural coercion occurs in many cases of community engagement [22]. This could be attributed to the use of influence by community leaders or government representatives in the communities who have been consulted by researchers, to compel community members using fear and intimidation to participate in research without obtaining the appropriate consent for the community members to get involved in the research.

Avoidance of exploitation by researchers is one key ethical issue regarding community engagement in research. Power imbalance between researchers and poorer communities leads to undue inducements pushing communities to participate research without fully understanding of risks and benefits, since they are attracted to monetary incentives that the project may come with, or from the fear of reprisal when they refuse to participate in the project [107]. CABs could serve as watchdogs in this area. Risks and benefits constitute principal ethical issues especially for biomedical researches that involve children or vulnerable groups like people with mental/brain health problems [108]. In this regard, researchers need to re-evaluate the need for such studies and also take the necessary measures to provide adequate information on research objectives, risks, and benefits to the representatives of these vulnerable groups before their engagement in [109]. Researchers also must ensure that research-related harm is avoided when engaging communities in research projects [110]. Regarding gender considerations in engaging communities in research, we noted that gender disparity issues mostly occur when it comes to the consent decision-making process for participation in research. This could be attributed to the structure of traditional patriarchal households where men are the heads therefore husbands of women are generally expected to make most decisions for the family, including negotiation of consent decisions to participate in research [111-113]. This particular gender-based issue should be taken into consideration by researchers when engaging communities in especially the research involving women. Researchers ought to ensure that research involving women also makes plans for their husbands´ involvement especially regarding consent. The men, for instance, should be informed of the study aims and possible outcomes as well as any risks and benefits to the study participant to ensure effective participation by the women. Proper communication with men will reduce resistance in allowing women to be part of research projects. Clear safe guards to men, as well as policies within the research group are necessary to ensure that they are “safe “in allowing women to participate in research. It is important to also properly communicate the scientific rationale of involving women in research in a language they can easily understand.

**Figure 2:**
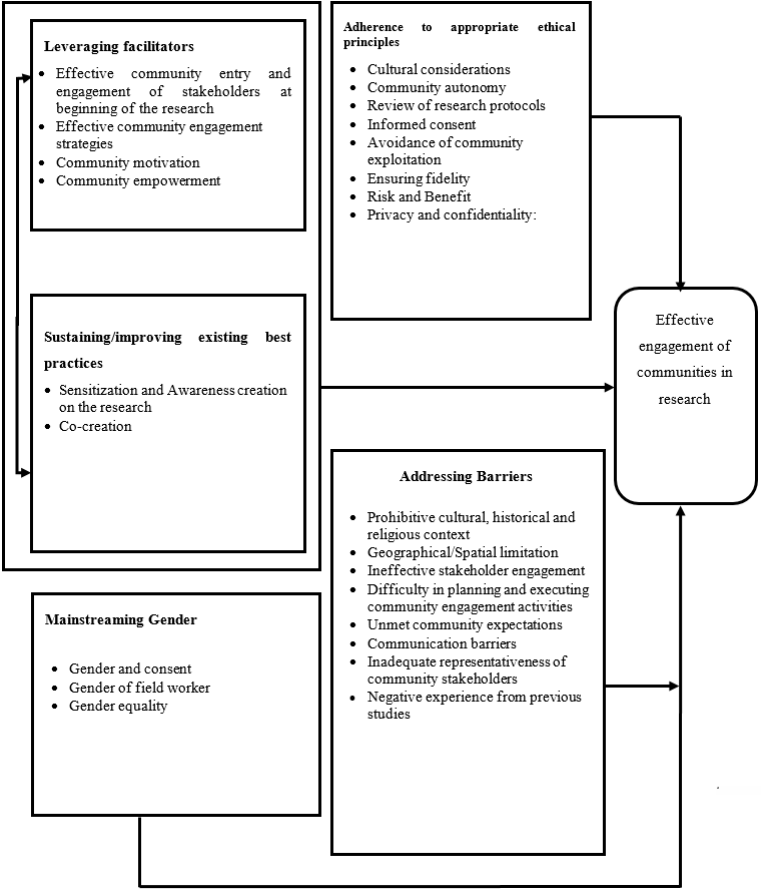
framework for effective engagement of communities in research

The gender of field workers has influence on the engagement process as it determines the extent to which participants feel free to express themselves [114,115]. Therefore, in most research involving sensitive information especially regarding women, measures need to be taken to ensure that research field workers are of the same gender. This could also help prevent field workers in research from involving opposite gender from exposure to sexual vulnerabilities. Concerning gender equality in community engagement, we realized from the reviews that both genders were involved at community engagement meetings despite the fact that women contribute less to such discussions. Hence, the need for researchers to engage the women separately to empower them to contribute to the engagements as most of the research projects are focused on them and their children. Education and communication on gender issues with men could facilitate active participation. Based on the key findings from our review, we propose a framework for effectively engaging communities in research (Figure 2).

### Strengths and limitations of the study

**Strengths of the study:** this review has ethical and gender considerations as central questions of the review. These issues have not been carefully assessed in previous reviews.

**Limitations of the study:** notwithstanding the outlined strengths, some limitations are imminent. First, excluding commentaries, personal reports and papers that have not been published in English or French may result in the loss of important findings. Secondly, some key papers published before 2000 might have been missed.

## Conclusion

While there are useful practices currently adopted in engaging communities in research in SSA, a number of barriers militates against such engagement efforts which negatively affect the implementation of community-based research projects. Gender mainstreaming and ethical standards are reported but not optimally operationalized. It is relevant to come up with clear ethics and gender frameworks to guide community engagement in research in SSA.

### What is known about this topic

- Meaningful community engagement is a major determinant of effective and meaningful research practice;
- Ethical and gender considerations are key in coming up with sustainable, inclusive and acceptable findings in research, relevant to evidence based health policy making;
- Context is an important consideration in effectively engaging communities in research.

### What this study adds

- Gender mainstreaming is abundantly reported as relevant, but inadequately operationalized in the community engagement in research process;
- Ethical considerations are not reported in a clear and systematic manner;
- We propose a 5 level framework for effective community engagement in research (leverage on facilitators, address barriers, adhere to context sensitive/responsive ethical standards, sustain/improve existing best practices, integrate gender mainstreaming in entire research cycle).

## Annexes

**Annex 1**: search strategy for Medline through OVID SP (PDF-162KB)

**Annex 2**: quality assessment of included papers (PDF-231KB)

**Annex 3**: single country studies (PDF-159KB)

**Annex 4**: multi country studies (PDF-169KB)

**Annex 5**: themes and sub-themes from our qualitative synthesis (PDF-222KB)
